# Supplementary material for: High-Light versus Low-Light: Effects on Paired Photosystem II Supercomplex Structural Rearrangement in Pea Plants
Source: Int J Mol Sci. 2020 Nov 16;21(22):8643. doi: 10.3390/ijms21228643 (PMC7698171; doi:10.3390/ijms21228643)
Supplement: Supplementary file 1 [file ijms-21-08643-s001.zip › Supplementary Materials.docx]

**SUPPLEMENTARY MATERIALS**

**High-light *versus* low-light: effects on paired Photosystem II supercomplex structural rearrangement in pea plants**

Alessandro Grinzato^1$^, Pascal Albanese^2,$,‡^, Roberto Marotta^3^, Paolo Swuec^4,5^, Guido Saracco^2^, Martino Bolognesi^4^, Giuseppe Zanotti^1^, Cristina Pagliano^2,^*

^1^Department of Biomedical Sciences, University of Padova, Via Ugo Bassi 58 B, 35121 Padova, Italy

^2^Applied Science and Technology Department–BioSolar Lab, Politecnico di Torino, Environment Park, Via Livorno 60, 10144 Torino, Italy

^3^Electron Microscopy Facility, Center for Convergent Technologies, Istituto Italiano di Tecnologia - IIT, Via Morego 30, 16163 Genova, Italy

^4^Department of BioSciences, University of Milano, Via Celoria 26, 20133 Milano, Italy

^5^Cryo-Electron Microscopy Facility, Human Technopole, Via Cristina Belgioioso 171, 20157 Milano, Italy

$ These authors contributed equally to this work

‡Present address: Biomolecular Mass Spectrometry and Proteomics, Bijvoet Center for Biomolecular Research and Utrecht Institute for Pharmaceutical Sciences, University of Utrecht, Padualaan 8, 3584 CH, Utrecht, The Netherlands

*Corresponding author: Cristina Pagliano

E-mail: cristina.pagliano@polito.it; Tel: +39 0110904626; Politecnico di Torino, Applied Science and Technology Department–BioSolar Lab, Environment Park, Via Livorno 60, 10144 Torino, Italy


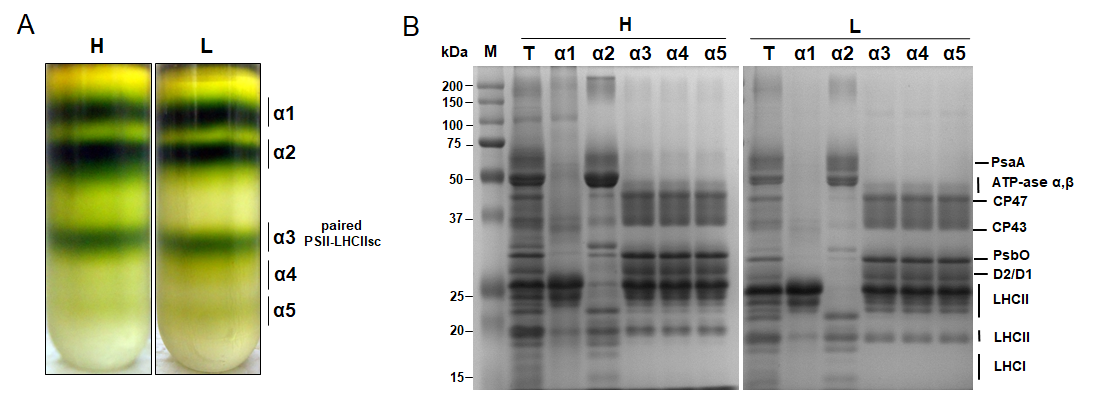


**Figure S1.** **Isolation and characterization of paired PSII-LHCII supercomplexes.** (**A**) Isolation of paired PSII-LHCII supercomplexes by sucrose gradient ultracentrifugation of thylakoid membranes extracted from pea plants grown at high-light (750 μmol m^−2^ s^−1^ photons, H) and low-light (30 μmol m^−2^ s^−1^ photons, L) and solubilized with α-DDM. (**B**) Coomassie stained SDS-PAGE of sucrose gradient bands α1-α5 isolated from thylakoids H and L solubilized with α-DDM and of corresponding sourcing thylakoid membranes (lanes T). The same amount of Chl (3 μg) was loaded on each lane. Labels on the left indicate the molecular weight positions (Bio-Rad precision plus). According to this separation, unpaired/broken PSII-LHCIIsc migrate in band α2, paired PSII-LHCIIsc in band α3 and paired PSII-LHCII megacomplexes in band α5. For a full biochemical description of these PSII-LHCII containing bands see our previous dedicated papers [1,2].


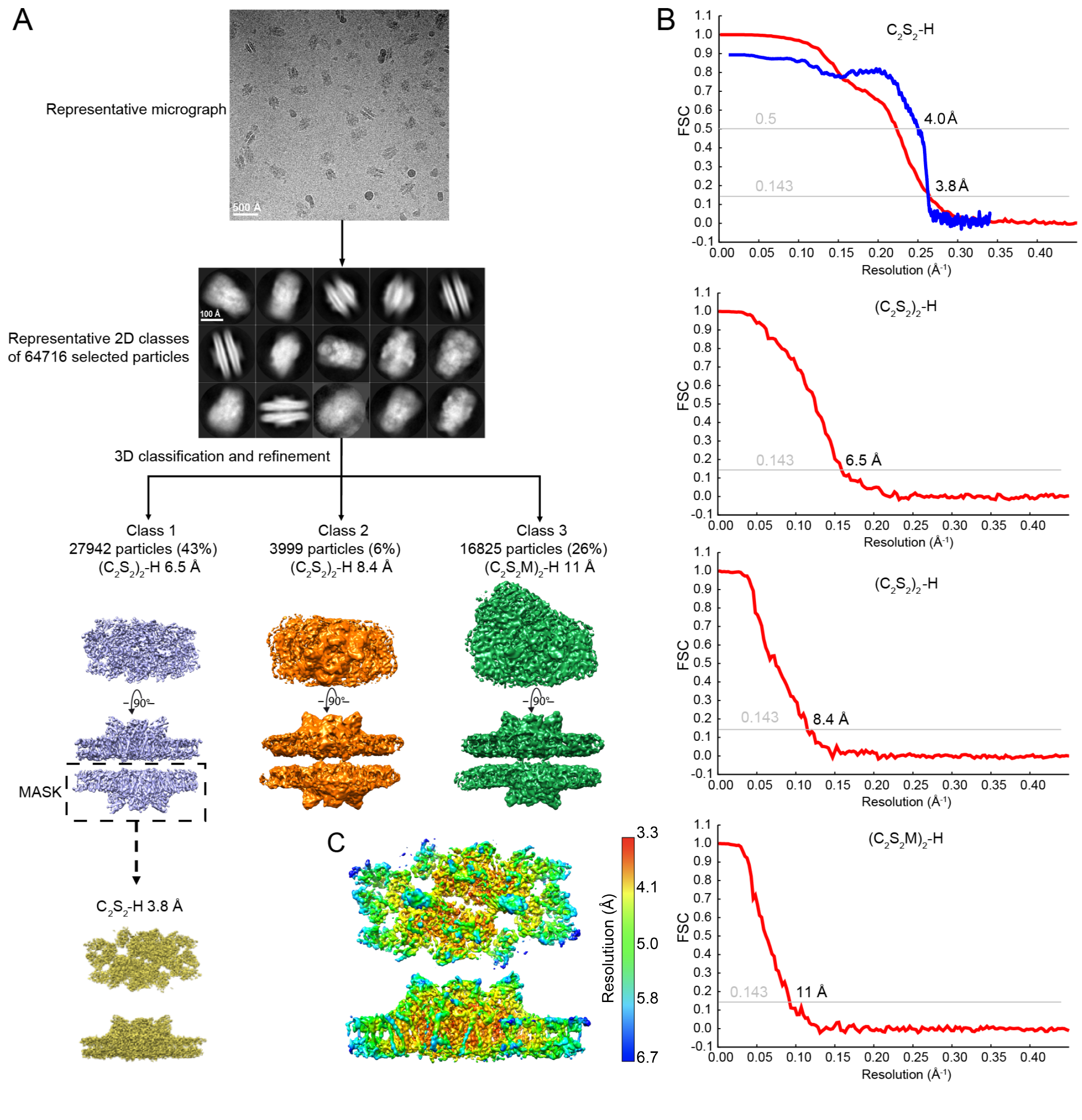


**Figure S2. Classification and refinement procedures used for image processing of the dataset of the high-light sample.** (**A**) Image processing workflow and (**B**) gold-standard Fourier Shell Correlation (FSC) curves of the density maps shown in Fig. 1 (gold standard FSC curves in red and map model FSC curve in blue). (**C**) Local resolution of the cryo-EM map of the C_2_S_2_-H supercomplex estimated by CryoSparc. For each map in panels A and C, the top view (top) shows the PSII from the lumenal side, normal to the membrane plane; the side view (bottom) is along the membrane plane.

**
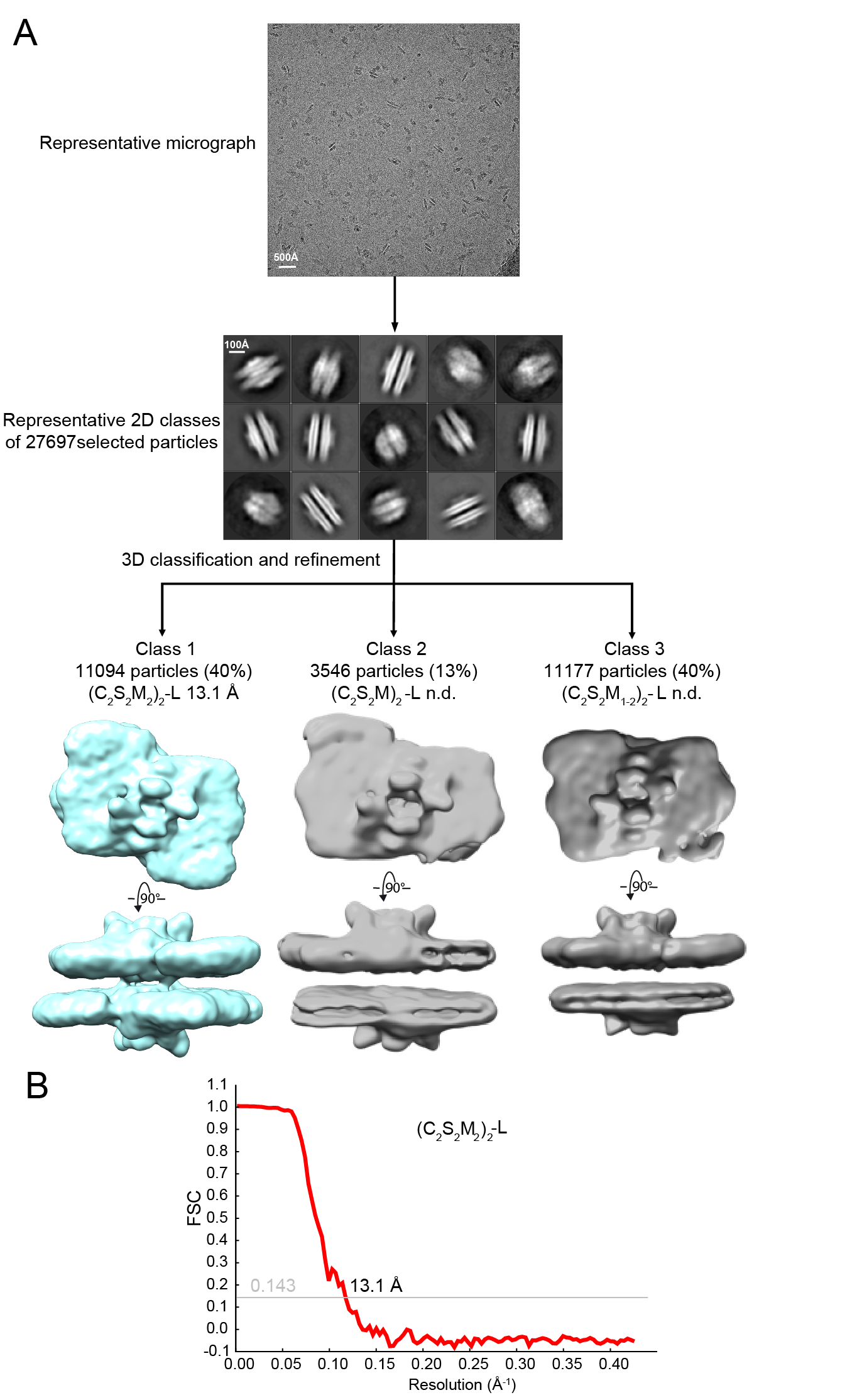
**

**Figure S3. Classification and refinement procedures used for image processing of the dataset of the low-light sample.** (**A**) Image processing workflow and (**B**) gold-standard FSC curve of the density map shown in Fig. 1 (FSC curve with criterion of 0.143). For each map in panel A, the top view (top) shows the PSII from the lumenal side, normal to the membrane plane; the side view (bottom) is along the membrane plane.


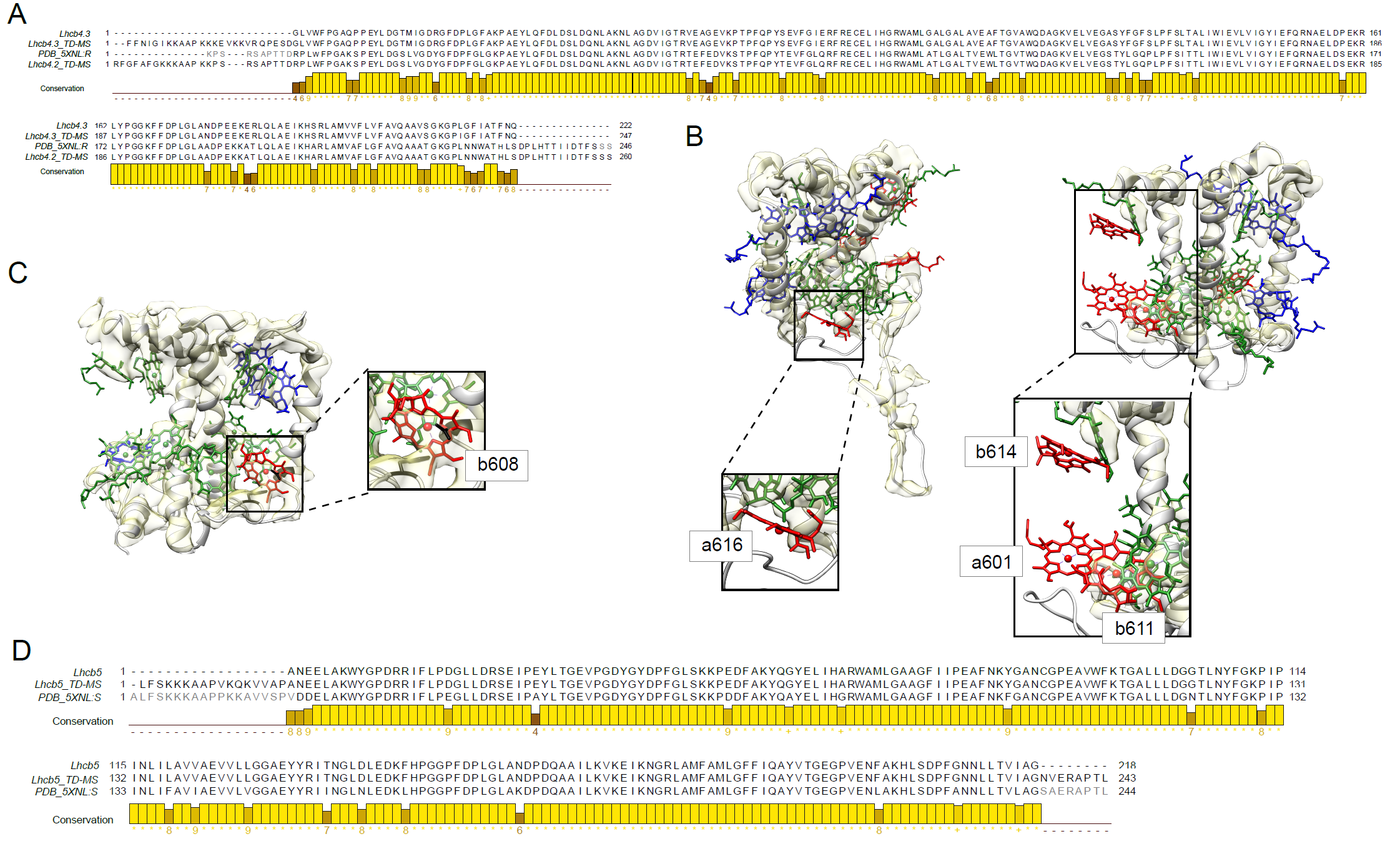


**Figure S4**

**Figure S4. Peculiar features of the Lhcb4.3 and Lhcb5 proteins of the C_2_S_2_ supercomplex isolated from plants grown in high-light.** Sequence alignment and corresponding density map of the protein Lhcb4.3 (**A** and **B**, respectively) and Lhcb5 (**D** and **C,** respectively). In each alignment, the first sequence corresponds to the amino acid residues inferred from the cryo-EM density map of the C_2_S_2_-H; the second is the sequence of the protein determined by top-down mass spectrometry (TD-MS) [3] on the paired PSII-LHCIIsc isolated from pea plants grown in the same high-light condition of this study; the third is the amino acid sequence of the Lhcb4 and Lhcb5 proteins present in the pea PSII-LHCIIsc structure from PDB 5XNL (chain R for Lhcb4 and chain S for Lhcb5) [4]. In the alignment in A, the fourth sequence is the sequence of the Lhcb4.2 protein determined by TD-MS [3] on the paired PSII-LHCIIsc isolated from pea plants grown in the same high-light condition of this study. In panels A and D, residues of the Lhcb4 (PDB 5XNL, chain R) and Lhcb5 (PDB 5XNL, chain S) proteins not resolved in the corresponding 3D map are coloured in grey. In panels B and C, representation respectively of the Lhcb4.3 and Lhcb5 proteins of the C_2_S_2_-H fitted inside their density map, with chlorophylls surrounded with a less defined or absent density map, but present in the 5XBL model, coloured in red.

**
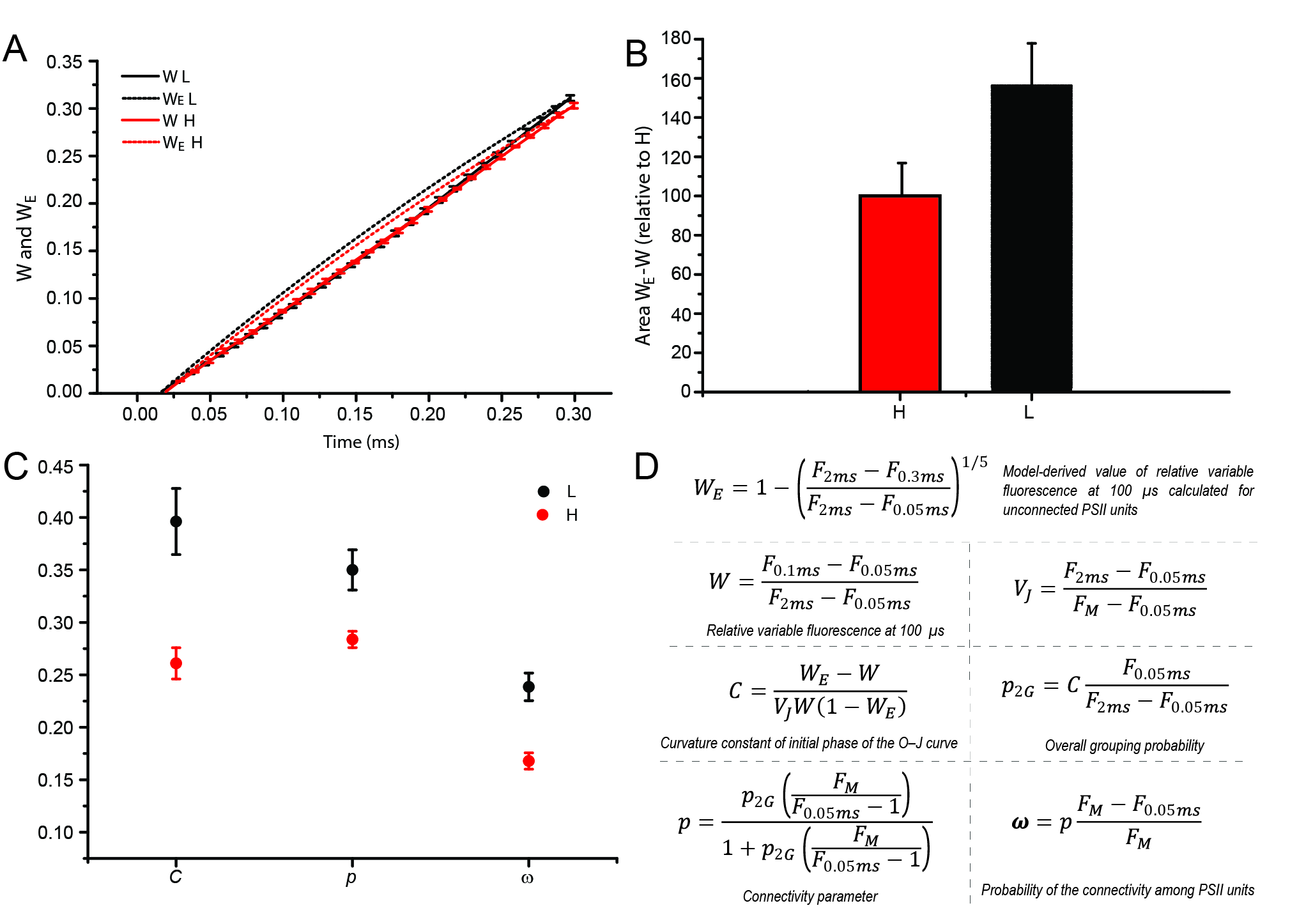
**

**Figure S5. Parameters derived from fast fluorescence kinetic measurements to estimate the energetic connectivity between PSII units of paired PSII-LHCII supercomplexes with different antenna size isolated from plants grown in high-light and low-light.** (**A**) The initial 300 μs from the OJIP transient shown in Fig. 5C replotted on a linear time scale for H and L samples. The normalized O–J phase of the OJIP curve is labeled W (solid line), and the theoretical exponential curve corresponding to the unconnected system is labeled W_E_ (dashed line). (**B**) Corresponding area under the curve of Fig. 5D. (**C**) Selected connectivity parameters and (**D**) their definitions derived from fast fluorescence kinetic measurements according to methods previously reported [5,6]. In panel C, all the parameters were significantly different between the two light conditions (p ≤ 0.05, derived from T-test, n=6).

| **Data collection** | **H dataset** | | | | **L dataset** |
| --- | --- | --- | --- | --- | --- |
| EM equipment | Titan Kryos | | | | Talos Artica |
| Voltage (kV) | 300 | | | | 200 |
| Detector | K2 | | | | Falcon III |
| Pixel size (Å) | 1.1 | | | | 1.82 |
| # of movie /# of frame | 2734/40 | | | | 1148/39 |
| Electron dose (e^-^/Å^2^) | 40 | | | | 50 |
| Defocus range (mm) | -1.2/-2.5 | | | | -0.5/-2.5 |
| **Reconstruction** | **C_2_S_2_**  **EMD-10865**  **PDB 6YP7** | **(C_2_S_2_)_2_**  **EMD-10866** | **(C_2_S_2_)_2_**  **EMD-10887** | **(C_2_S_2_M)_2_**  **EMD-10867** | **(C_2_S_2_M_2_)_2_**  **EMD-10868** |
| # of particles | 27942 | 27942 | 3999 | 16825 | 11094 |
| Resolution (Å)  GS FSC (0.143) | 3.8 | 6.5 | 8.4 | 11 | 13.1 |
| Map Model FSC (Å)  (0.143/0.5) | 3.8/4.0 |  |  |  |  |
| CC (mask) | 0.72 |  |  |  |  |
| Chains | 72 |  |  |  |  |
| Total atoms / hydrogens | 71784/0 |  |  |  |  |
| Residues | Protein 7002  Ligand 325  Water 0 |  |  |  |  |
| Clashscore | 9.13 |  |  |  |  |
| MolProbity score | 2.07 |  |  |  |  |
| Ramachandran plot (%)  Favored  Allowed  Outliers | 95.21  4.79  0 |  |  |  |  |
| Rotamer outliers (%) | 2.15 |  |  |  |  |

**Supplementary Table 1.** Cryo-EM data and overall statistics for C_2_S_2_ model (MolProbity)

**Supplementary References**

[1] P. Albanese, M. Manfredi, A. Meneghesso, E. Marengo, G. Saracco, J. Barber, T. Morosinotto, C. Pagliano, Dynamic reorganization of photosystem II supercomplexes in response to variations in light intensities, Biochim. Biophys. Acta - Bioenerg. 1857 (2016) 1651–1660. https://doi.org/10.1016/j.bbabio.2016.06.011.

[2] P. Albanese, J. Nield, J.A.M. Tabares, A. Chiodoni, M. Manfredi, F. Gosetti, E. Marengo, G. Saracco, J. Barber, C. Pagliano, Isolation of novel PSII-LHCII megacomplexes from pea plants characterized by a combination of proteomics and electron microscopy, Photosynth. Res. 130 (2016) 19–31.

[3] P. Albanese, S. Tamara, G. Saracco, R.A. Scheltema, C. Pagliano, How paired PSII–LHCII supercomplexes mediate the stacking of plant thylakoid membranes unveiled by structural mass-spectrometry, Nat. Commun. 11 (2020) 1361. https://doi.org/10.1038/s41467-020-15184-1.

[4] X. Su, J. Ma, X. Wei, P. Cao, D. Zhu, W. Chang, Z. Liu, X. Zhang, M. Li, Structure and assembly mechanism of plant C_2_S_2_M_2_-type PSII-LHCII supercomplex, Science 357 (2017) 815–820. https://doi.org/10.1126/science.aan0327.

[5] R.J. Strasser, A.D. Stirbet, Estimation of the energetic connectivity of PS II centres in plants using the fluorescence rise O–J–I–P. Fitting of experimental data to three different PS II models., Math. Comput. Simul. 56 (2001) 451–462. https://doi.org/10.1016/s0378-4754(01)00314-7.

[6] A. Stirbet, Excitonic connectivity between photosystem II units: What is it, and how to measure it?, Photosynth. Res. 116 (2013) 189–214. https://doi.org/10.1007/s11120-013-9863-9.
